# Status of trace metals and arsenic in sediments and catfish muscles (*Clarias gariepinus*) from the Eastern Tanzanian basin

**DOI:** 10.1371/journal.pone.0306335

**Published:** 2024-08-29

**Authors:** Edward Mayila, Alexanda Mzula, Cyrus Rumisha, Martine Leermakers, Filip Huyghe, Marc Kochzius

**Affiliations:** 1 Department of Microbiology, Parasitology and Biotechnology, College of Veterinary Medicine and Biomedical Sciences, Sokoine University of Agriculture (SUA), Morogoro, Tanzania; 2 Marine Biology – Ecology, Evolution & Genetics, Biology Department, Vrije Universiteit Brussel (VUB), Brussels, Belgium; 3 Department of Animal, Aquaculture and Range Sciences, Sokoine University of Agriculture (SUA), Morogoro, Tanzania; 4 Analytical, Environmental and Geochemistry (AMGC), Vrije Universiteit Brussel (VUB), Brussels, Belgium; Fisheries College and Research Institute, INDIA

## Abstract

Trace metals and metalloids are groups of chemical elements that naturally occur in low concentrations and cycle in the environment driven by natural processes and human activities. They have a persistent and bio-accumulative tendency in the environment, and certain trace metals and metalloids have become a public health concern. This study assesses the concentration of eleven trace metals and a metalloid in sediments and catfish muscle from five study sites in the Eastern Tanzanian River basin. Forty catfish tissues and fifteen sediment samples were collected and analyzed using ICP-MS. Concentrations of As, Cd, Co, Pb, and Zn did not exceed the United States Environmental Protection Agency (USEPA) guideline for pollution of sediments, while Al Cr, Al, Mn, and V with values ranging from (118.54 to 70154.55) indicating moderately polluted. The stations Java-Sadaani and Matandu showed the highest Cr, Ni, and Cu concentrations, but the potential ecological risk index (RI) was low (RI < 95). In the catfish muscle tissue, the levels of Cd, Pb, Cu, and Zn did not surpass the EU and FAO/WHO limits and results ranged from 2.22 to 35.22mg/kg. Low levels of accumulation of Cd, Pb, and As were found in this study compared to catfish muscles from other studies, whereas the concentrations of other trace metals and metalloids analyzed had comparable results. Biota/sediment accumulation factors (BSAF) were all < 1. The weekly metal intake (MWI) results ranged from 6.89E-04 to 2.43E+01 μg/know-1week-1, indicating a low risk as the value did not exceed the FAO/WHO established Permissible Tolerable Weekly Intake (PTWI). The non-carcinogenic health risk result THQ was 4.43E-02 and the carcinogenic health risks result HI was 4.42E-05 which indicated tolerable levels of risks as both the values of the Target Hazard Quotient (THQ) and the Hazard Index (HI) was < 1, and the carcinogenic target risk (TR) is < 0.0001. The highest TR values were observed for Cr and Ni. We recommend a continued monitoring of the changes in trace metal levels in the environment and biota together with continuous public health education on the dangers of high levels of trace metals.

## 1. Introduction

Trace metals and metalloids are natural constituents of the Earth’s crust [1]. They are released into the environment through natural processes, such as the weathering of minerals [2, 3], volcanic eruptions [4], and atmospheric deposition [5]. Anthropogenic activities related to land use, such as mining, sewage sludge disposal, land clearing by burning, industrial discharge, application of pesticides, and source material used in the manufacturing of micronutrient and inorganic fertilizers, equally release trace metals in the environment [6–8]. When released in sufficient quantities, these trace metals and metalloids can lead to environmental pollution, contamination of biota, and risks to human health [9].

Sediment pollution by trace metals has been reported in many African countries. The common metal pollutants include lead (Pb), cadmium (Cd), mercury (Hg), copper (Cu), cobalt (Co), zinc (Zn), chromium (Cr), nickel (Ni), manganese (Mn), iron (Fe), vanadium (V), and metalloid arsenic (As). The main sources of metal pollution in Africa vary from oil spills in the west, solid municipal waste in most parts of the continent, as well as mining activities in the eastern and southern parts [10]. In Tanzania, a profile for contaminants in fluvial sediments of a rural and urban drainage basin in Tanzania was done [7]. Trace metals and a metalloid were also detected in vegetables grown around the Msimbazi and Sinza rivers in Dar es Salaam [11] and were recorded on the quality of water and sediments as an impact of mining and farming in the Mara River basin [12]. Trace metals and metalloids in sediments of rivers in Dar es Salaam were reported with enrichment of Cd, Sn, Pb, Co, Cr, and Zn [13], and in mangrove sediments along the Indian Ocean coast [14]. A study in Morogoro [15] reported trace metals and arsenic in catfish from sewage ponds, while trace metals in aquaculture, and found high levels of trace metals in catfish and tilapia in Morogoro and Arusha [16].

Trace metals and arsenic can be toxic even at low concentrations. They are highly stable, persistent in the environment, and bioaccumulate in the food chain [9, 17, 18] including agricultural products such as sauces [19] coconut milk [20], vegetables [11], wheat-based sweets [21] and in roadside plants and soils [22]. Bioaccumulation occurs when the rate of uptake and storage of an element is higher than the rate at which it can be metabolized, excreted, or in any other way neutralized [23, 24]. Fish and other aquatic animals can accumulate large amounts of certain trace metals through the ingestion of suspended particles during feeding [25]. They can also accumulate trace metals by constant ion exchange processes of dissolved metals across lipophilic membranes like the gills and adsorption of dissolved metals on tissue and membrane surfaces [12]. High concentrations of trace metals become toxic and exert harmful effects on fish. Trace metals and metalloids can accumulate through the food chain, eventually reaching humans and other top predators [26].

The USEPA has listed priority pollutant trace metals to be monitored in food items due to their danger to human health. The list includes Cd mercury (Hg), Cu, Co, Fe, Ni, Pb, and Mn [27]. The accumulation of trace metals and metalloids has been reported in both fishes of freshwater and marine origin, with catfish *Clarias spps* and tilapia *O*. *niloticus* being the notable species from the freshwater reported to have trace metals [28]. They have also been found in skipjack tuna (*Katsuwonus pelamis*) in Sri Lanka [29] and the tissue of swordfish (*Xiphias gladius)* collected in the Mediterranean Sea [30]. A study in the Xincun Lagoon of China found that higher trophic levels at the bottom of the sea were significantly higher than in lower trophic and pelagic levels [31]. Studied species included *Acipenser sinensis*, *Trachurus japonicus*, *Cynoglossus sinicus*, *Tamnaconus hypargyreus*, *Chirocentrus dorab and Pecapterus maruadsi*. Other species were *P*. *anomala*, *L*. *brevironstris*, *U*. *bensasi*, *G*. *flamentosus*, *S*. *chinensis*, *N*. *gronovii*, *B*. *novae-zeelandiae*, *and A*. *argentatus* [31].

Regular consumption of contaminated catfish with trace metals and metalloids is also known as potentially harmful. The known negative effects include lethal and/or chronic diseases, involving neurological signs [32], Alzheimer’s, dementia, hepatic syndrome [24], cardiovascular conditions, skin conditions, systemic conditions, and renal failure [1]. Other conditions are liver diseases, developmental disabilities, autism, cerebral palsy, and bone disorders. At a cellular level, elevated trace metal and metalloid concentrations can cause disruption of cellular functioning, damage-repairing processes, and apoptosis [33].

The combined health risk due to consumption of catfish contaminated with several trace metals and metalloids needs to be assessed. This is because multiple metals may increase the carcinogenic risk [34]. Certain trace metals and metalloids accumulate faster in certain organs and increase health effects on certain organs compared to others. For instance, Cr and Ni have pronounced effects in respiratory organs; Cd specifically affects the kidneys and intestine [35, 36], Pb is associated with neurotoxicity and nephrotoxicity, while As intake through drinking water can cause cancer of the urinary bladder [17, 35, 36]. Compounds of Cr, Cd, and Ni have been listed as group 1 human carcinogens [36].

The African sharp-tooth catfish (*Clarias gariepinus)* is a commercially important freshwater fish [37], it is second to tilapia in Tanzania. Initially, it was found mainly in the tropics but now it has been imported to many parts of the globe [38]. It is an invasive species on other fish species [39]. They are easily cultured, reproduce large numbers of fingerlings, and can grow to a large size even above 5kg. Catfish are omnivorous fish, feeding on plant material, plankton, arthropods, mollusks, other fish, reptiles, and amphibians [40]. In Tanzania, catfish are more abundant during the rainy season, when the rivers overflow and breeding takes place. The fish are occasionally sold raw, but most often, they are smoked for preservation or fried and sold along the roadside. Because of their habitat, (bottom-dwelling) and their elevated position (at the apex) of the food web [39], they are prone to bioaccumulation and form a potential health risk if trace metal concentrations in rivers become too high.

Whereas studies have reported contamination of sediments and catfish tissues with trace metals and metalloids; in Tanzania, catfish is the second fish species among freshwater fish consumed owing to the increased aquaculture activities. There is a need to assess the status of contamination in catfish caught from the study area and estimate the health risk that the catfish-consuming community may face due to exposure to a complex mixture of pollutants. This study assessed the concentration and distribution of trace metals and arsenic in sediments and catfish tissues by ICP-MS and judged following the USEPA and EU guidelines. This was an attempt to provide information on the quality of sediment to biota and its level of contamination in catfish. To our knowledge, there is little information currently addressing this topic in the study area. The objectives of this study were to determine the concentrations of eleven trace metals and arsenic in sediments and catfish muscles from five areas within the west Indian Ocean basin of Tanzania and hence, the pollution status and the potential ecological and health risks. Risk indices such as chronic daily intake (CDI), estimated daily intake (EDI) for a lifetime period, target hazard quotient (THQ), and hazard index (HI) from consumption of catfish contaminated with trace metals were measured to characterize the non-carcinogenic and carcinogenic health risks. Sampling sites were selected considering the catfish-consuming community, the availability of catfish, and the likely treatments conducted at the sources of the rivers. The possible causes of metal contamination are also discussed below. The results of this study inform on the pollution status and the risks to the catfish-consuming community caught from the study area.

## 2. Materials and methods

### 2.1 Study area

**Fig 1:**
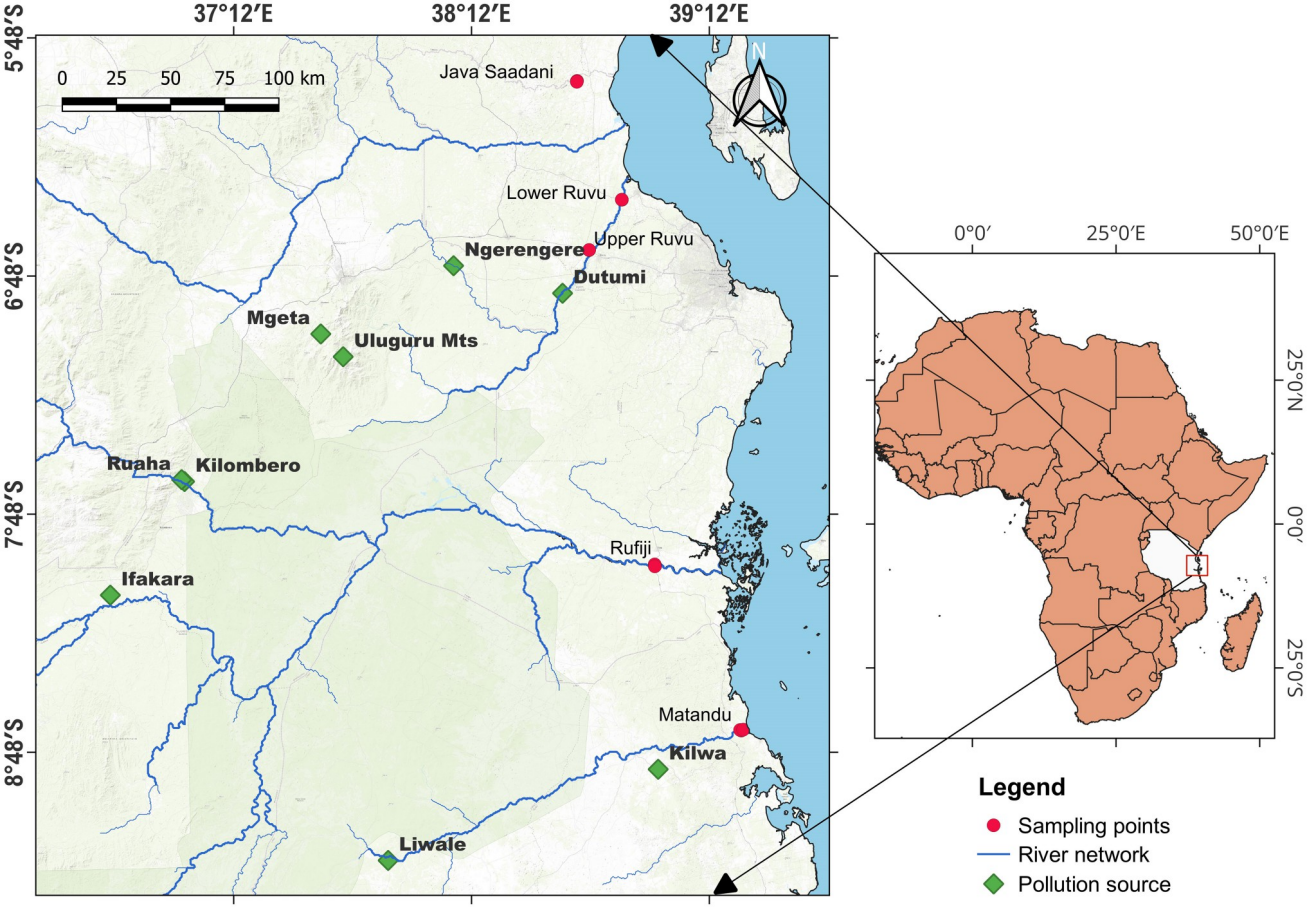
An extract of the map of Tanzania showing the main rivers (blue) and sampling points (red dots). The numbers near the sampling sites are the sampled stations. Data source: Environmental Systems Research Institute (ESRI) provider for ArcGIS, and United States Geological Survey) USGS.

Five sample sites were studied in three different rivers flowing into the Western Indian Ocean (WIO). Two stations were sampled at Ruvu River (upper and lower), one at each of the Rufiji and Matandu sites, and a pond at Java village near Saadani National Park (Fig 1).

Rufiji is the largest river entirely within Tanzania, flowing to the WIO from Ruaha, Kilombero, and Ifakara sources (Britannica Encyclopaedia, 2010; World Atlas). The Kilombero area is known for its large estates of sugar cane plantations and the largest sugar factory in the country, and the Ifakara area has numerous rice plantations. These farming and factory activities might contribute to trace metal pollution in this river through the application of pesticides and inorganic and micronutrient fertilizers [41].

Matandu is a short river flowing through the Lindi region from uphill in the Liwale districts passing through an extended lowland near the Rufiji basin to Kilwa towards the Indian Ocean [42]. It is about 100 km away, South of Rufiji River, with little human activity and low population density. Since there are no large plantations and estates around Matandu, the expected trace metal pollution could come from within the short distances that the river has travelled.

The Java-Saadani Pond is located within the Ruvu Wami basin, near Saadani National Park, which has a low human population density, again with a few human activities and expected low levels of trace metal pollution.

The Ruvu River, from its sources of the Uluguru mountains through Ngerengere, Mgeta, and Dutumi points, runs and crosses the Kibaha and Bagamoyo main roads. These are Tanzania’s busiest main roads, linking the North of the country with the East and South to the neighboring countries [43]. The area contains several petrol stations, some at a distance less than a kilometer apart.

### 2.2 Sampling and types of samples

Samples were collected at the end of December 2021 and the beginning of January 2022. Three sediment samples, each 50g, were collected at each of the five sites. Sediments were collected at a depth of about 10 cm and a distance of about 50 meters apart. Each of the collected samples was mixed using a wooden stick and a portion of about 50g of the well-mixed sediment samples were collected in a falcon tube. Fifteen sediment samples were collected.

Catfish were bought from fishermen close to the respective study sites, making sure they were caught at the indicated sampling points. A piece of muscle tissue weighing 5–10 grams was cut from eight catfish from each of the sites using a surgical blade. Forty catfish muscle samples in total were collected. No anesthetic agent was used as samples were taken from dead catfish.

### 2.3 Preparation

#### 2.3.1 Preparation of catfish tissue samples

Fish muscle tissues were dried in a hot air oven (Beger, Slovenia) at 50°C for five consecutive days and then re-weighed to calculate the water content (S1 Table). Dried tissues were ground into fine particles, packaged into small plastic bags, and kept in a refrigerator until transport to the laboratory for trace metal analysis. About 0.1–0.15g of each of the tissue samples was weighed and placed into Teflon tubes for digestion. Certified reference material (ERM-CE278, mussel tissue), as well as a blank solution, were included in each digestion batch as a negative control for QA/QC check. Five milliliters of distilled concentrated nitric acid (65% Fisher Chemicals, Pittsburgh, Pennsylvania, USA) were added into each of the Teflon tubes, containing the weighed tissues and heated to 180°C (ramp to 180°C for 10 minutes and held at 180 °C for 10 minutes) in a microwave oven (Anton Paar Microwave Go). After the digestion step, 40 ml of deionized water (Milli-Q water, Millipore) was added, and samples were transferred to pre-labeled plastic tubes.

#### 2.3.2 Preparation of sediment samples

Sediment samples were dried in an oven (Beger, Slovenia) overnight at a temperature of 70°C. Dry sediment was ground into fine particles using a mortar and pestle and was digested in a microwave oven (Anton Paar Microwave Go) using a tri-acid mixture (nitric acid, hydrofluoric acid, and hydrochloric acid). About 0.1–0.15g of the ground samples were placed into Teflon tubes for acid digestion. Reference material (LGC6139, river clay sediment) and a blank were digested along with each batch as a negative control for QA/QC check. The digestion process involves two steps. In the first step, 5ml concentrated distilled nitric acid HNO_3_ (69%, Fisher Chemicals, Pittsburgh, Pennsylvania, USA—pro analysis), 4 ml hydrofluoric acid (47–51%, Fisher Chemicals, Pittsburgh, Pennsylvania, USA, trace metal grade), and 2ml hydrochloric acid HCl (37%, Fisher, chemicals, Pittsburgh, Pennsylvania, USA trace metal grade) was added to the dried material and heated to 180°C within 10 minutes, followed by a hold time of 10 minutes at 180°C. After digestion, the vessels were cooled to room temperature, and then 30ml of boric acid (4% Fisher Chemicals, Pittsburgh, Pennsylvania, USA) was added into each tube to complex the excess hydrogen fluoride to prevent the danger of explosion and corrosion in the equipment. The samples were again heated in the microwave oven to 120°C (10 minutes ramp time to 120°C, 5 minutes hold at 120°C) and transferred into a pre-labelled plastic tube for analysis. Between each digestion, the Teflon tubes were rinsed with Milli-Q water, followed by an acid-cleaning procedure in the microwave oven. Ten ml HNO_3_ (Fisher, Chemicals, Pittsburgh, Pennsylvania, USA pro analysis) were added to each tube and heated in the microwave oven to 180°C (10 minutes ramp to 180°C, 5 minutes hold at 180°C). The tubes were rinsed with Milli-Q water and allowed to dry in a hood with laminar airflow.

#### 2.3.3 Laboratory analysis of trace metals

An Inductive Coupled Plasma–Sector Field Mass Spectrophotometer (ICP-MS; Thermo Scientific Element II, USA) was used to analyze trace metals both in sediments and in catfish tissues. Digested samples were further diluted ten times before analysis. Calibration was performed by dilution of a multielement standard (Merck Multielement standard XIII) and Indium (In), because of its chemical stability in blends of dilutions, was used as an internal standard. The elements Al, As, Cd, Co, Cr, Cu, Fe, Mn, Ni, V, Pb, and Zn were quantified at different resolutions low, medium, and high accordingly. The results obtained for the certified reference material were all within the certified range and hence sample analysis preceded proper QA/QC results Interim sediment quality guidelines (ISQGs) were used to decide the level of pollution, with the threshold effect level (TEL) referred to as the concentration below which the minimal effect range within adverse effects rarely occur, while probable effect levels (PELs) as the mean concentration above which the probable effect range within adverse effects frequently occur. The reference values of sediment quality guidelines for pollution characterization of trace metals in mg/kg were adopted from USEPA (1977) [44], and the Canadian Council of Ministers of the Environment (CCME), ISQL, and PEL (CCME, 1999) [45, 46].

### 2.4 Assessment of contamination and ecological parameters

#### 2.4.1 Enrichment factor (EF)

The Enrichment Factor (EF) was calculated using the universally adopted background concentration values for the upper continental crust for areas without known background data [47]. These values, expressed in μg/g, are Al (77,440), As (2), Cd (0.1), Co (12), Cr (35), Cu (14), Fe (30,890), Mn (527) Ni (1944), V (53), Pb (17), and Zn (52).

The EF for each trace metal was calculated by normalizing against Al as a reference metal because it is a highly conservative and an essential constituent of clay minerals [48]. The EF was computed using the formula below:

$$\text{EF}=\;\left(\frac{\text{Cm}}{\text{Cref}}\right)\text{sample}/\;\left(\frac{\text{Cm}}{\text{Cref}}\right)\text{background}$$

where EF is the enrichment factor, Cm is the concentration of the metal, and Cref is the concentration of the reference metal (Al) in the sample and the background values [49–51]. Results for EF were categorized as follows: EF up to 1 = no enrichment; 3–5 = minor enrichment; 5–10 = moderately severe enrichment; 10–25 = severe enrichment; 25–50 = very severe enrichment, while an EF above 50 is considered extremely severe enrichment [51, 52].

#### 2.4.2 Contamination factor (Cf) and contamination degree (Cd)

Cf is the concentration of a trace metal in the sediment divided by the background value [53] expressed as

Cf=Cmetalsample/Cmetalbackground

However, for a better application in pollution assessment, Hakanson [53] proposed an alternative measure called the degree of contamination (Cd). This is calculated as the sum of the Cf for each sample expressed as:

$$Cd=\overset{i=n}{\underset{i=1}{\sum }}Cfi$$

Results for Cf were interpreted following Hakanson and Maanan et al. [53, 54] as Cf < 1 = low contamination; 1–3 = moderate contamination; 3–6 = considerable contamination; and > 6 = very high contamination. The degree of contamination (Cd) results were interpreted following Hakanson [53] When the value is less than the total number of elements considered under evaluation (in this case, 6), it indicates a low degree of contamination, 6–12 is moderate, 12–24 is considerable, and > 24 is a high degree of contamination, indicating serious anthropogenic pollution.

#### 2.4.3 Assessment of the potential ecological risk index

The pollution and ecological risk indices (CF, EF, Igeo, PLI, and PERI) were assessed using the method referred in [53, 55, 56]. The potential ecological risk index (RI) assesses the degree of risk that trace metal pollution in sediment presents to biota, which depends on the toxicity of trace metals and the response of the environment to contamination. To achieve this, the following equations were applied:

$$\begin{array}{l} RI=\sum_{i=1}^{i=n}E\;r^{i}\\ Er^{i}=T^{i}rC^{i}f \end{array}$$

RI is the sum of risk factors for trace metals in sediments, Er^i^ is the potential ecological risk factor for a given trace metal (i), and Cf is the contamination factor. The T^i^r is the toxic response factor, representing the potential hazard of trace metal contamination by indicating the toxicity of a particular trace metal under specific environmental conditions. The standardized T^i^r values for Cd, Cr, Cu, Ni, Pb, and Zn were respectively given as 30, 2, 5, 5, 5, and 1 by Hakanson [53].

Six trace metals (Cd, Pb, Cr, Ni, Cu, and Zn) were assessed for their ecologic risk potential. Risk index (RI) values were classified depending on the risk level as follows: < 95 indicates a low risk, 95–190 = moderate ecological risk, 190–380 = considerable ecological risk, and > 380 = very high ecological risk [53].

#### 2.4.4 Geoaccumulation (lgeo index) and pollution load index (PLI)

These parameters determine and define heavy metal contamination in sediments by comparing current concentrations with pre-industrial levels. The Igeo index was calculated and interpreted by following the equation given in [55, 57], using the formula.


$$\text{lgeo}={\text{log}}_{2}\left(\text{Ci}/1.5*\text{Bn}\right).$$

lgeo is the geoaccumulation index, Ci is the concentration of the trace metal in the sample, 1.5 is a constant figure given as 1.5, Bn is the background value of the metal, and log_2_ is the logarithm of the number to the base of 2.

The classification of contamination based on the lgeo results followed the lgeo class and the lgeo index as lgeo < 0, belongs to index class 0, and is translated as uncontaminated; 0 < lgeo <1 is index class 1, implying uncontaminated to moderately contaminated; 1< lgeo < 2, index class 2, refers to moderately contaminated, 2 < lgeo < 3 is index class 3 meaning moderately to heavily (strongly) contaminated, 3 < lgeo < 4 is index class 4 meaning heavily (strongly) contaminated, but 4 < lgeo < 5 is index class 5, meaning heavily (strongly) to extremely contaminated while lgeo > 5 is index class 6 extremely contaminated.

The pollution load index, (PLI), was calculated using the formula

$$\text{PLI}=\sqrt[{n}]{\left(\text{Cf}1.\text{Cf}2.\text{Cf}3\ldots .\text{Cfn}\right)}$$

Cf1,2,3…n refers to contamination factor to element 1,2,3..n and n is the number of elements under consideration. PLI values greater than 1 indicate polluted sediment, while below 1, suggests unpolluted sediment.

#### 2.4.5 Bioaccumulation factor

The biota-sediment accumulation factor (BSAF) was also calculated to assess the accumulation of trace metals in catfish tissues from the associated sediment by relating the concentrations of the trace metals in the sediments with those in the catfish tissues.


BSAF=Concentrationintissue/Concentrationinsediment

Bioaccumulation was categorised into three groups according to the BSAF values, as suggested by [51, 58] as follows < 1 = deconcentrator; 1–2 = microconcentrator; > 2 = macroconcentrator.

### 2.5 Assessment of potential human health risk due to consumption of catfish

#### 2.5.1 Estimations of weekly intake

To evaluate the health risks, the concentration of trace metals in the catfish tissues (dry weight) was converted into concentrations in wet weight using the following formula:

$$Trace\;metal\;conc\;in\;wet\;weight=\left(100-\%\;water\right)xConc.\;in\;dry\;tissue$$

The trace element concentrations were compared with accepted limits for human consumption established by the European Commission, US-EPA, and WHO FAO [59–61]. Trace metal weekly intake was calculated for the elements Cd, Cr, Cu, Ni, Pb, and Zn, and compared with provisional tolerable weekly intakes (PTWI). The PTWI per kilogram body weight (bw) is 2.5μg, 23.3μg, 500μg, 11μg, and 70μg respectively, for Cd, Cr, Cu, Ni, and Zn. The previously established PTWI of 25μg/kgbw^-1^ for Pb is no longer accepted by WHO and the European Food Safety Authority (EFSA) since EFSA identified neurotoxicity cardiovascular effects and nephrotoxicity in children as critical effects for risk assessment [62].

The weekly metal intake was estimated based on the available local data on the overall average annual fish consumption, i.e. 8.5 kg/person [63], and converted to average fish consumption per week (AfCW). However, catfish is the second most consumed freshwater fish after tilapia owing to the aquaculture production practices.


$$\text{AfCW}\left(\frac{\text{Per}\;\text{capita}\;\text{consumption}\;\left(\text{kg}/\text{person}\right)}{\text{Number}\;\text{of}\;\text{days}\;\text{in}\;\text{consumption}/\text{year}\left(365\right)}\right)\text{x}7\;$$

The average fish portion consumed per week (AfP) was estimated as

AfP=AfCWx0.5

considering that only half of the total weight of the fish is edible [2].

The trace metal weekly intake (MWI) in μg/kg BW for an average person of 70 kg was then calculated according to [64], using the following formula:

MWI=AfP×MC

Whereby AfP is the average fish portion consumed per week and MC is metal concentration in catfish tissue expressed in μg/g wet weight.

#### 2.5.2 Assessment of non-carcinogenic and carcinogenic health risk

The Target Hazard Quotient (THQ) of non-carcinogenic and Target Risk (TR) for carcinogenic risk indicators were calculated and compared to the US standards [65]. The Chronic Daily Intake (CDI) expressed in mg/kg BW/day over a lifetime was calculated using the following formula:

$$\text{CDI}\left(\text{ing}\right)=\frac{\text{C}\;\text{x}\;\text{EF}\;\text{x}\;\text{ED}\;\text{x}\;\text{IRF}}{\text{Atn}\;\text{x}\;\text{bw}}$$

Where C is the trace metal concentration in catfish (μg/g ww), ED is the exposure duration (70 years), EF is the exposure frequency (365 days/year), and IRF is the rate of ingestion of fish per day (11.6 g/day), bw is the body weight for an adult person (70 kg) and Atn–is the average exposure time which for a lifetime is the same as (EF x ED) [28, 66–68].

*2*.*5*.*2*.*1 Non-carcinogenic health risk*. For non-carcinogenic health risk evaluation, the target hazard quotient was calculated as:

$$\text{Target}\;\text{Hazard}\;\text{Quotient}\left(\text{THQ}\right)=\text{CDI}/\text{RfD}$$

Where the metal Chronic Daily Intake (CDI) and the Reference Dose (RfD) are all expressed in μg/kg bw/day. The reference dose is an allowable dose below which no non-carcinogenic health effects should result from a lifetime of exposure. The RfD proposed by Zheng et al. [69] was adopted. For Pb, there is no safe RfD. The THQ is the universally accepted risk index from [65], for estimating the risk of food contaminants [65, 70]. A THQ value below 1 indicates low chances of non-carcinogenic health effects, while a THQ value above 1 suggests a risk of non-carcinogenic effects, with the likelihood increasing as the THQ value increases. The overall potential risk of non-carcinogenic effects of the combined effect of the trace elements, the hazard index (HI) was calculated as per [71].


HI=∑THQ

*2*.*5*.*2*.*2 Carcinogenic health effects*. Cancer risk assessments are expressed as individual lifetime carcinogenic target risk (TR). This assessment is defined as the daily average intake per kilogram of body weight, multiplied by an element-specific factor, the so-called Slope Factor (SF). Since long-term exposures of experimental animals to low doses of hazardous substances are difficult to evaluate, the SF is derived from the approach that high-dose exposure, over a brief period, is equivalent to low-dose exposure over a lifetime. It expresses the probability of an individual developing cancer because of exposure to a carcinogenic substance. Risks for carcinogens of one on one hundred thousand (10^−5^) are acceptable, but TR should not exceed 1 on ten thousand (10^−4^) [65], calculated as:

$$\text{Carcinogenic}\;\text{target}\;\text{risks}\left(\text{TR}\right)=\text{CDI}/\text{SF}$$

Whereby the Chronic Daily Intake dose (CDI) (mg/kgbw/day) and slope factor (SF) in (mg/kg bw/day)^-1^.

### 2.6 Statistical data analysis

Results were checked for normality and homoscedasticity using the Shapiro-Wilk normality test, which relied on the skewness and kurtosis values. Sediment data were normally distributed and results were presented in tables showing mean values and standard deviations and analyzed by one-way Analysis of Variance (ANOVA) using GraphPad Prism version 9.0.0 software (Dotmatics, 2020). Otherwise, tissue sample results were analyzed using Kruskal-Wallis ANOVA by ranks as the data were non-normally distributed, and results were presented in a table with mean, median, and SD while correlation analysis was performed on Microsoft Excel window 10 software.

### 2.7 Ethical statement

This study was conducted with approval from the Tanzania Fisheries and Aquaculture Research Technical and Ethical Committee (TaFReTEC) under Ref. No: TAFIRI/HQ/RES.CLEARANCE/ VOL/11/341. Catfish tissues and sediments were sampled following ethical guidelines and regulations set forth by TaFReTEC to ensure the responsible and ethical conduct of research.

## 3. Results and discussion

### 3.1 Trace metals in sediment samples

Trace metals and arsenic concentrations varied at each of the study sites are presented in Table 1. The concentration with their respective sediment quality guidelines is shown in Table 2. The mean concentrations in sediments in μg/g was dominated by Al and Fe given at decreasing concentrations as Al (70155), Fe (41779), Mn (789), Cr (121), V (119), Zn (66), Ni (51), Cu (31), Co (21), As (2.56) and Cd (0.12). The concentrations of As, Cd, Co, Pb, and Zn did not exceed the US EPA guideline and hence did not pollute the sediment. The sediment was moderately polluted with Al, Cr, Fe, Mn, and V. Threshold effect levels (TEL) of Cr and Cu were exceeded at all sample sites. Samples collected from Java-Saadani and Matandu showed the highest Cr and Cu concentrations. Java-Sadaani also shows the highest Ni concentrations. The concentrations of Cr and Ni were significant between sites (P<0.05). Java-Saadani is within the junction of Wami-Ruvu and the Pangani basin, in which a study reported higher levels of Ni in the Pangani basin [7]. This could be an extension of the metal ore or rather a run-off effect of the metal from a far source of the Pangani basin to Java. The high levels of Al and Fe can be attributed to the fine grain size of the sediments, and further, the two metals are among the major components of the continental crust [47]. Therefore, they are usually in higher concentrations than other trace metals.

**Table 1:** Trace metal concentration (mean ± SD, n = 3) in sediments (in μg/g) from the five sample sites.

Metal	Java-Saadani	Upper Ruvu	Lower Ruvu	Rufiji	Matandu
Al	69145 ± 3710	75438 ± 15193	60835 ± 7840	79961 ± 16401	65392 ± 11115
As	3.23 ± 1.08	2.75 ± 1.75	1.67 ± 0.34	3.36 ± 0.24	1.82 ± 0.44
Cd	0.11 ± 0.02	0.136 ± 0.02	0.13 ± 0.04	0.10 ± 0.01	0.14 ± 0.04
Co	25.98 ± 5.62	24.32 ± 4.37	15.95 ± 3.35	18.42 ± 5.71	18.32 ± 6.14
Cr	190.02 ± 37.22	102.37 ± 21.64	76.01 ± 10.69	103.92 ± 6.25	131.86 ± 60.20
Cu	41.77 ± 8.27	27.91 ± 7.62	24.85 ± 5.50	28.12 ± 9.23	31.65 ± 12.11
Fe	49659 ± 9881	46028 ± 9772	31886 ± 7912	45646 ± 10724	35675 ± 12295
Mn	594 ± 115.01	1372 ± 934.86	599 ± 281.20	980 ± 511.37	396 ± 100.32
Ni	84.96 ± 18.13	43.93 ± 10.28	30.92 ± 5.98	38.92 ± 9.67	55.50 ± 28.46
V	142.98 ± 25.8	129.48 ± 15.9	95.46 ± 20.6	116.96 ± 26.7	107.80 ± 35.4
Pb	17.04 ± 1.95	15.35 ± 0.38	14.13 ± 1.38	20.11 ± 6.34	16.01 ± 1.89
Zn	72.39 ± 14.91	75.15 ± 17.49	61.64 ± 29.15	66.44 ± 10.16	53.95 ± 20.29

**Table 2:** Sediment quality guideline values by USEPA and CCME (mg/kg).

Status	Unpolluted	Moderate polluted	Polluted (up to)	Heavily polluted	CCME TEL	CCME PEL
Al			18600*			
As	<3		6	>8	5.9	17
Cd			3	>6	0.6	3.5
Co			70**			
Cr	<25	25–75	75	>75	37.3	90
Cu	<25	25–50	50	>50	18.7	103
Fe	<17000	17000–25000	25000	>25000		
Ni	<20	20–50	50	>50		
V			60			
Mn	<300	300–500	500	>500		
Pb	<40	40–60	60	>60	35	91
Zn	<90	90–200	200	>200	123	315

Key:

* [54] and

** [72], USEPA–United States Environmental Protection Agency, CCME–Canadian Council of Ministers of the Environment.

### 3.2 Comparison to other studies

The present findings were compared with results from other studies performed on sediments using methods with a similar principle reported results in the same units (mg/kg or μg/g) as per the present study. The results are summarised in Table 3. In the Okumeshi River of Nigeria, reported higher values of Cd than from all sample sites of this study using a flame absorption spectrometer, but low levels of Pb, Cr, and Ni [73]. Another study at Kubanni River in Nigeria with an atomic absorption spectroscopy reported higher concentrations of Cd, Cu, and Zn than the present study, but comparable values for Pb, and low mean values for Cr, Mn, and Ni [24]. Trace metal levels in sediments of the Tanoe River of Bono in Ghana using a graph furnace method [74], had lower concentrations of all the assessed metals compared to the present study except for Cd, while a study in Lake Naivasha of Kenya reported comparable levels to this study for Co and Cu, higher levels of Fe, Mn Pb, and Zn, but a lower value of Cr [75] using using energy-dispersive X-ray fluorescence. A study in Ethiopia reported polluted sediments with higher mean values for As (623 μg/g), Cd (151 μg/g), and Cr 375 (μg/g) [76] than the present study using inductively coupled plasma optical emission spectrometry (ICP-OES). This area of Ethiopia, however, is vulnerable to pollutants from different activities, including laboratory wastes, medical wastes, garages, and car washing, untreated municipal effluent, agricultural non-point source discharges, and animal feedlots within Jimma in Ethiopia [76].

**Table 3:** Comparison of mean trace metals in sediments μg/g of this study to other studies.

Metals	Eastern Tanzania	Omukeshi, Nigeria.	Tano, East Bono, Ghana	Kubanni, Nigeria.	Naivasha, Kenya	Awetu, Ethiopia.
Al	70154.2					
As	2.566		1.38			623
Cd	0.124	1.32	2.44	4.65		152
Co	20.6				24.04	
Cr	120.836	0.87	6.73	29.23	27.44	375
Cu	30.86		1.67	52.43	33.74	
Fe	41778.4				53655	
Mn	788.4			40.41	1506	
Ni	50.846	1.21		19.94		
Pb	16.528	0.45	6.34	16.98	22.11	2006
V	118.536					
Zn	65.916		18.1	79.12	231.94	
Ref.	Present study	[73]	[74]	[24]	[75]	[76]

### 3.3 Enrichment factor

**Fig 2:**
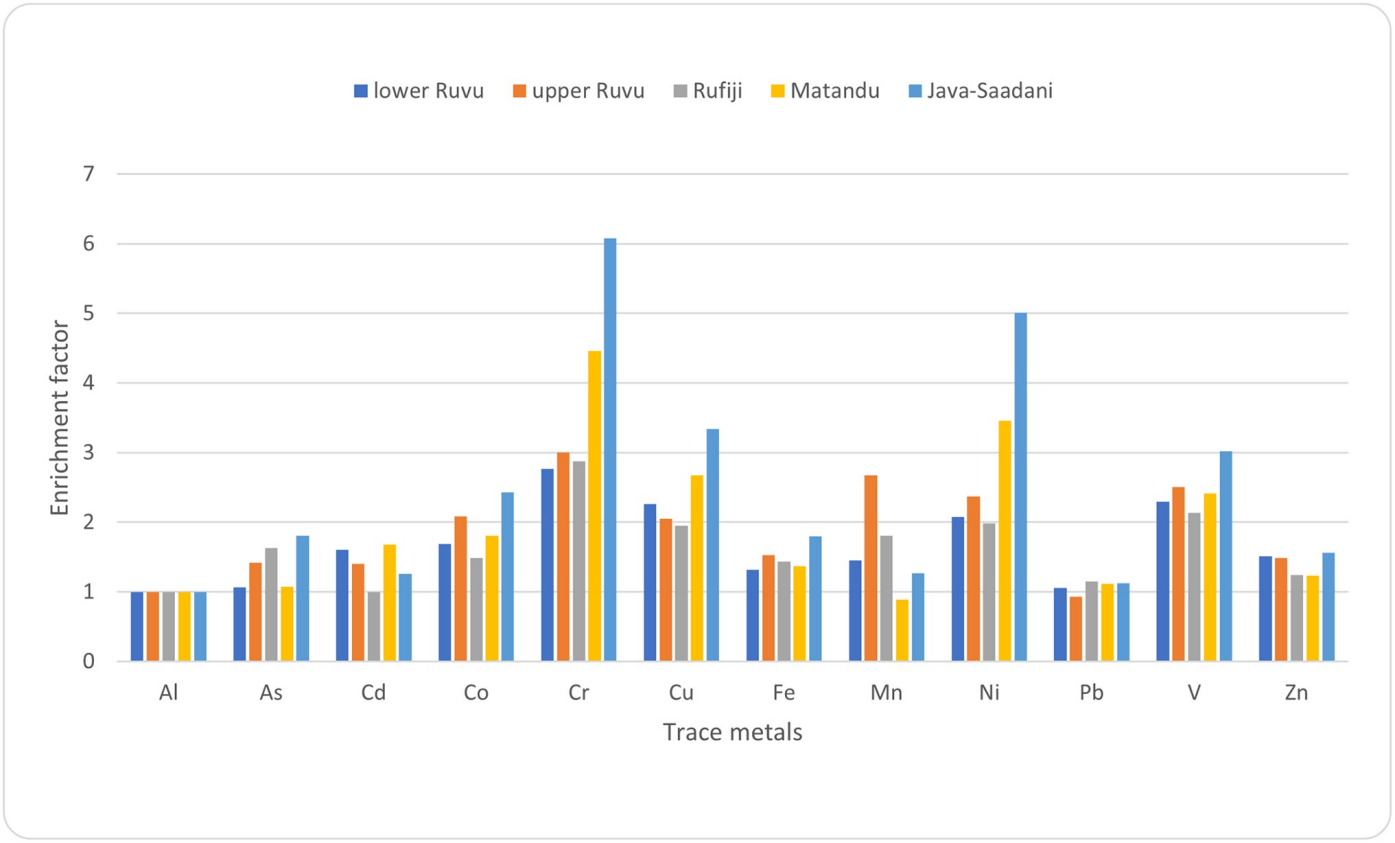
Enrichment factors of sediments with trace metals results at different study sites. Interpretation: EF up to 1 = no enrichment; 3–5 = minor enrichment; 5–10 = moderately severe enrichment; 10–25 = severe enrichment; 25–50 = very severe enrichment, while an EF above 50 is considered extremely severe enrichment.

Results for EF are presented in Fig 2. Cr and Ni showed the highest enrichment factor, especially in Java-Saadani and Matandu, but contributed to a minor to moderately enriched sediment. Also, Cu and V showed a higher enrichment in Java-Saadani and Matandu. Most of the sediment in the study areas was still within the average natural abundance for the elements assessed.

### 3.4 Correlation analysis

The correlations among the elements are shown in Table 4. High correlations (with*) significant at P < 0.05, were observed between Ni and Cr (0.98*), Co and V (0.97*), V and Fe (0.96), Cr and Cu (0.85*), Co and Fe (0.92), Ni and Cu (0.92*), Al and Fe (0.80) as well as Al and Mn (0.81). Fe correlated positively with every trace metal except Cd, and Cd correlated only (negatively) with As. Al showed a weak correlation with Cr, Ni, and Cu, but correlated well with the rest of the trace metals.

**Table 4:** The correlation between trace metals from sediments of the study sites.

Metal	Cd	Pb	Al	V	Cr	Mn	Fe	Co	Ni	Cu	Zn	As
**Cd**	1.00											
**Pb**	-0.14	1.00										
**Al**	-0.12	0.70	1.00									
**V**	-0.16	0.47	0.68	1.00								
**Cr**	-0.10	0.18	0.22	0.75	1.00							
**Mn**	-0.20	0.32	0.81	0.51	-0.02	1.00						
**Fe**	-0.30	0.56	0.80	0.96	0.65	0.65	1.00					
**Co**	-0.13	0.37	0.62	0.97*	0.72	0.55	0.92	1.00				
**Ni**	-0.09	0.25	0.28	0.81	0.98*	0.05	0.70	0.80	1.00			
**Cu**	-0.13	0.44	0.46	0.82	0.85*	0.23	0.76	0.80	0.92*	1.00		
**Zn**	-0.37	0.21	0.64	0.80	0.45	0.65	0.83	0.79	0.51	0.63	1.00	
**As**	-0.52	0.36	0.64	0.66	0.53	0.63	0.79	0.64	0.48	0.53	0.65	1.00

Key:

*Indicate R results with a significant difference (P<0.05).

### 3.5 Potential ecological risk factors (Er^I^) and risk index

Twelve elements Al, As, Cd, Co, Cr, Cu, Fe, Mn, Ni, Pb, V, and Zn were assessed for their contamination factor and contamination degree, while six trace metals (Cd, Pb, Cr, Ni, Cu, and Zn) were assessed for their ecologic risk potential, pollution load index, and geoaccumulation index. In this regard, the results for the parameters contamination factor (Cf) and contamination degree are presented in Table 5. Results indicate that the environment is considerably contaminated with Cr (Cf 3–6), and moderately contaminated with Ni, V, Cu, Co, Mn, Fe, As, Zn, and Cd (Cf 1–3). The trace metals Pb, Al, V, and Zn had a low contamination factor (Cf < 1).

**Table 5:** Results for contamination factor and ecological risk indices.

Contamination factor and degree				Ecologic risk	
Trace metal	Mean (μg/g)	Background (μg/g)	Cf	Tr	Er^i^ (Cf*Tr)
Al	70154.55	77440	0.91		
As	2.57	2	1.28		
Cd	0.12	0.1	1.24	30	37.12
Co	20.6	12	1.72		
Cr	120.84	35	3.45	2	6.9
Cu	30.86	14	2.2	5	11.02
Fe	41778.9	30890	1.35		
Mn	788.82	527	1.5		
Ni	50.85	19	2.68	5	13.38
Pb	16.53	17	0.97	5	4.86
V	118.54	53	2.24		
Zn	65.92	52	1.27	1	1.27
Degree of Contamination (Cd)			20.8	RI	74.56

Note: Cf = Contamination factor, Tr = Toxic response, Er^i^ = Ecologic risk factor; RI = l Risk index. Result interpretations: CF: <1 = low contamination; 1–3 = moderate contamination; 3–6 = considerable contamination; and >6 = very high contamination.

For Ecological risk, Er^i^ < 40 indicates a low ecological risk; 40–80 is a moderate ecological risk; 80–160 is a considerable ecological risk; 160–320 is a high ecological risk and Er^i^ >320 is a very high ecological risk. For the risk index (RI) values were classified as follows: < 95 indicates a low risk, 95–190 = moderate ecological risk, 190–380 = considerable ecological risk, > 380 = very high ecological risk.

The overall potential ecological risk index (RI = 74) indicates a low ecologic risk. Individual Er^i^ values for all assessed trace metals indicate a low risk, with Cd having an Er^i^ value of 37.12 that exceeds all other trace metals, but still with a low risk to biota. The individual trace metals pollution load index (PLI) results for the six assessed metals were 1.24, 3.45, 2.2, 0.97, and 1.27, respectively, for Cd, Cr, Cu, Ni, Pb, and Zn, while the overall PLI was 1.77 which indicate pollution of sediments as PLI exceeds 1.

The lgeo results are presented in Table 6. The findings suggest uncontaminated to moderately contaminated sediments, with most of the assessed elements having an lgeo index class ranging from 0–2, and Ni (with lgeo value −6) least polluted sediments in all of the study sites. The sources of these contaminants are mainly natural activities indicated by negative values of the lgeo class in metals. The current concentration of trace metals in sediment poses a lesser risk to the biota. These findings are in agreement with other findings reported in the Rufiji [77] but differ from the results of moderate to considerable risk reported from rivers in the industrialized city of Dar es Salaam [64]. Considering sediment pollution status with individual trace metals, we can generally say that most of the assessed trace metals and metalloids do not pollute the sediment in the studied areas, but the PLI parameter indicates pollution.

**Table 6:** The geoaccumulation of the sites and overall mean concentrations of the various trace metals and arsenic.

Element	Java -Saadani	Upper Ruvu	Lower Ruvu	Rufiji	Matandu	Mean Igeo	Interpretation of Igeo classes		
Al	-0.75	-0.62	-0.93	-0.54	-0.83	-0.73			
As	0.11	-0.13	-0.85	0.16	-0.72	-0.23			
Cd	-0.45	-0.14	-0.21	-0.58	-0.10	-0.28	Index class	lgeo value	Level of contamination classification
Co	0.53	0.43	-0.17	0.03	0.03	0.19	0	lgeo <0	Uncontaminated
Cr	1.86	0.96	0.53	0.99	1.33	1.20	1	0 < lgeo <1	Uncontaminated to moderately contaminated
Cu	0.99	0.41	0.24	0.42	0.59	0.56	2	1 < lgeo <2	Moderately contaminated
Fe	0.10	-0.01	-0.54	-0.02	-0.38	-0.15	3	2 < lgeo <3	Moderately to heavily (strongly) contaminated
Mn	-0.41	0.80	-0.40	0.31	-1.00	0.00	4	3 < lgeo <4	Heavily (strongly) contaminated
Ni	-5.10	-6.05	-6.56	-6.23	-5.72	-5.84	5	4 < lgeo <5	Heavily (strongly) to extremely contaminated
V	0.85	0.70	0.26	0.56	0.44	0.58	6	lgeo >5	Extremely contaminated
Pb	-0.58	-0.73	-0.85	-0.34	-0.67	-0.63			
Zn	-0.11	-0.05	-0.34	-0.23	-0.53	-0.24			
Mean	-0.25	-0.37	-0.82	-0.46	-0.63	-0.46			

The geoaccumulation of the sites lgeo index class values of the study values and interpretation

### 3.6 Trace metals and arsenic in catfish tissue samples

The mean concentrations (with median and SD) of trace metals and arsenic in catfish tissues are presented in Table 7. The mean concentrations of results in decreasing order (in μg/g dry weight) were Fe (89.19), Al (46.90), Zn (35), Cu (2.22), Mn (2.01), Cr (1.32), Ni (0.55), Co (0.14), V (0.12), As (0.06), Pb (0.04), and Cd (0.003). The concentrations of Pb, V, Cr, Mn, Co, Ni, and Zn varied significantly between sites (P<0.05). Iron, Zn, and Cu were the most abundant essential transition elements, forming integral parts of proteins involved in many biological functions. At high concentrations, however, they are toxic, binding to protein molecules they are not destined to by displacing original metals from their natural binding sites and causing malfunctioning of cells [78]. Metal homeostasis is generally maintained through balancing adsorption and excretion [79]. Also, Cr, Mn, and Co are essential elements, whereas Al, Ni, V, As, Pb, and Cd have no metabolic function for the fish. In Table 8, the concentrations measured in the catfish were compared to the maximum permissible values set for some elements by the EU and WHO/FAO. The reported maximum permissible levels (expressed in wet weight) were recalculated to dry weight based on the computed catfish water content of about 80% (S1 Table) in the fish muscle tissue. The measured concentrations of Cd, Pb, Cu, and Zn were all lower than the maximum permissible levels. Cr and Ni showed an inverse relationship between the concentrations in fish and sediments. The highest concentrations of Cr and Ni in sediments were found in Java-Saadani and the lowest in lower Ruvu, whereas the catfish of Java-Saadani showed the lowest concentrations and those from lower Ruvu the highest. The highest concentrations of Al in catfish were also observed in lower Ruvu, whereas for the other elements, no significant differences between the stations were found.

**Table 7:** Mean concentration (with maximum and minimum) dry weight (in μg/g, n = 8) of trace metals in catfish (*Clarias gariepinus*) tissue sampled at study sites.

Site	Statistics	Cd	Pb	Al	V	Cr	Mn	Fe	Co	Ni	Cu	Zn	As
Matandu	Mean	0.003	0.04	39.28	0.13	1.66	1.79	83.20	0.21	0.67	2.33	37.29	0.05
	Median	0.003	0.04	45.55	0.14	1.69	1.85	86.38	0.20	0.67	2.46	38.96	0.04
	stdev	0.001	0.01	14.40	0.04	1.15	0.61	32.49	0.11	0.69	0.52	8.87	0.04
Java-Saadani	Mean	0.001	0.03	41.88	0.12	0.17	1.50	73.76	0.12	0.17	1.94	39.58	0.07
	median	0.001	0.03	29.34	0.09	0.17	1.12	66.36	0.11	0.19	1.64	40.72	0.04
	stdev	0.000	0.02	29.14	0.08	0.09	0.87	38.91	0.06	0.05	0.59	3.53	0.08
Lower Ruvu	Mean	0.004	0.05	81.10	0.19	3.34	2.24	154.12	0.18	0.97	2.54	32.53	0.05
	Median	0.004	0.05	86.50	0.20	3.84	2.18	131.29	0.14	0.73	2.32	32.72	0.04
	stdev	0.001	0.01	27.85	0.07	1.90	0.64	65.19	0.11	0.82	0.94	6.38	0.03
Rufiji	Mean	0.003	0.05	50.14	0.09	0.81	3.02	70.34	0.08	0.45	1.83	27.94	0.03
	Median	0.003	0.05	40.60	0.09	0.76	2.49	67.50	0.09	0.40	1.64	28.11	0.03
	stdev	0.001	0.01	30.00	0.04	0.53	1.46	26.32	0.03	0.23	0.76	3.87	0.01
Upper Ruvu	Mean	0.002	0.02	22.08	0.09	0.63	1.49	64.52	0.11	0.49	2.45	38.75	0.10
	Median	0.002	0.02	18.27	0.06	0.19	1.34	49.39	0.11	0.37	2.23	37.60	0.07
	stdev	0.001	0.01	14.22	0.06	0.77	0.55	32.41	0.03	0.38	0.59	9.33	0.07

Key: Some maximum permissible limits with their sources * [80] ** [81] Cd-0.25*, Cu-150**, Pb- 1.5*, and Zn-500**. These values were recalculated to dry weight, assuming 80% water content S1 Table.

**Table 8:** Comparison of mean results of trace metals (μg/g dry weight) in catfish (*Clarias gariepinus*) muscle tissue to other studies.

Metal / Regions	Tanzania	Mozambique	Okumeshi, Nigeria	Brazil	Aligarh, India	Vaal, S. Africa	Central Ethiopia
Al	46.9					27–41	
As	0.06	12.66		17			
Cd	0.003	1.15	0.63				0.12
Co	0.14				6.81		
Cr	1.32		0.06	0.62	<LOD	0.16	9.02
Cu	2.22	18.9		1.17	22.7	1.7–3.4	ND
Fe	81.9	168.5			295.4	41–78	62.42
Mn	2.01		1.97		4.54	0.75–1.58	42.52
Ni	0.55		0.17	0.28	34.1	0.3–8.5	4.06
Pb	0.04	3.65	<0.01			0.5–5.67	4.03
V	0.12						
Zn	35.22	44.22		31	75	17–40	19.79
Reference	Present study	[84]	[73]	[85]	[86]	[82]	[83]

Key: <LOD–below the Limit of Detection

### 3.7 Comparison of trace metals in catfish tissues with other studies

The mean values for the concentrations (μg/g dry weight) of trace metals and arsenic in catfish muscles of the present study were compared to concentrations of trace metals in catfish muscles obtained from other studies using comparable methods that report concentrations in the same units. Aluminium concentrations are comparable with those found in catfish of the Vaal River, South Africa, measured by Varian Atomic Absorption Spectrophotometer (SPECTRA AA-10) [82]. In the present study, we found higher concentrations of Fe and Zn than in the Akaki River catchment in central Ethiopia using an inductively coupled plasma optical emission spectrometer, ICP-OES, (Agilent 700 Series, USA) [83], but lower values of Cd, Cr, and Mn. The mean Pb of 0.04 in the present study was lower than the mean Pb values of 4.03 and 3.64 reported in Ethiopia [83, 84] in the muscles of a catfish from rivers in Mozambique measured by inductively coupled plasma optical emission spectroscopy (ICP-OES) (Model ICPE–9820, Shimadzu Corporation, Japan). The level of Cd (0.003) in the present study was also lower than the mean (0.63) reported from the Okumeshi Delta River in Nigeria [73] and far less than the mean (1.15) reported in Mozambique. The mean As results (17) from catfish in Brazil measured by an optical emission spectrometer with inductively coupled plasma [85] exceeded the mean findings of As in Mozambique and was by far above the mean As results of the present study. Our report of Mn was almost equal in concentrations to the results from Okumeshi in Nigeria [73] but lower than that reported in India with an Atomic Absorption Spectrophotometer (Perkin Elmer AA800) [86]. The concentration of Fe and Cu in Mozambique was higher than all reported results except in India (Table 8). The concentration of Zn reported in Bayelsa state, Nigeria [87] using the AAS equipment exceeds the level reported elsewhere in the studies under comparison, while that of Brazil compares to the present study. The concentrations of Cr and Ni are generally higher than those observed in other areas, except for the very high value reported for Ni in Aligarh, India [86]. A study in central Ethiopia [83], did not detect any Cu but reported higher levels of Cd, Cr, Mn, and Ni than the present study and lower concentrations of Fe and Zn.

### 3.8 Bioaccumulation factor

**Fig 3:**
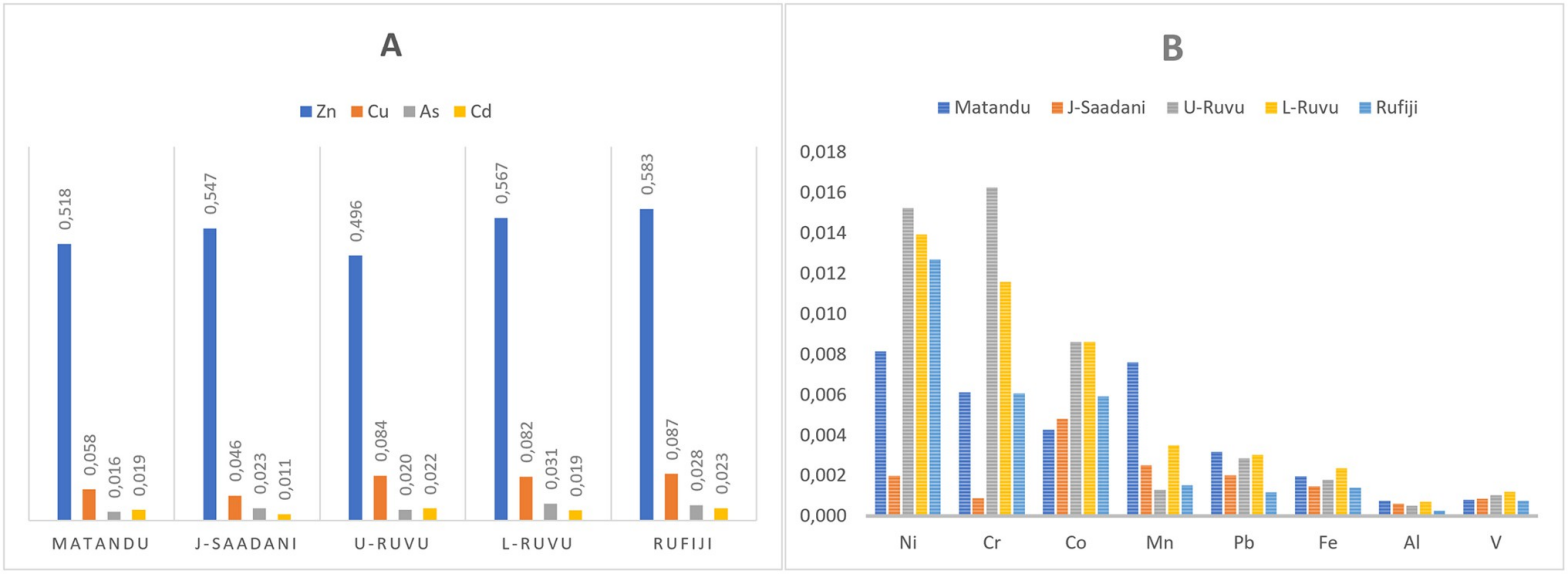
Biota sediment accumulation factor for Zn, Cu, As and Cd (A) and Ni, Cr, Co, Mn, Pb, Fe, Al and V (B) Matandu, Java-Saadani, Ruvu (upper and lower) and Rufiji. The BSAF is categorized into three categories: <1 = deconcentrator; 1–2 = microconcentrator, and >2 = macroconcentrator.

The biota-sediment accumulation factor (BSAF) results for trace metals assessed were all below 1. Generally, tissues from all five sites did not accumulate trace metals. Results were, however, significantly different between sites (P < 0.0001), with Zn exceeding the BSAF of all other trace metals studied in the present study, followed by Cu (Fig 3). These metals, Zn and Cu are known essential elements performing necessary functions as coenzymes in the formation of tissues and organs [88]. The obtained BSAF is based on the most edible part, the catfish muscles and results could vary if other tissues were assessed because trace metals accumulate differently in different fish tissues and essentially accumulate stronger in the liver, gills, and kidney [89].

### 3.9 Assessment of potential health risk

#### 3.9.1 Estimations of weekly intake

The average trace metals and arsenic concentrations in catfish expressed in μg/g wet weight and the estimated trace metal/metalloid weekly intake (MWI) for an average person of 70kg and an average weekly fish consumption of 163 g are shown in Table 9 and compared to provisional tolerable weekly intakes (PTWI). As can be seen, the MWI values are much lower than the PTWI (generally more than 100 times). The PTWI for Pb has been withdrawn [62], but the MWI is ten times lower than the benchmark dose for toxicological effects, however, the benchmark doses of 0.5, 1.5, and 0.63 μg/kgbw were effective for developmental neurotoxicity, systolic blood pressure, and on chronic kidney disease respectively [62].

**Table 9:** Estimated trace PTWI and MWI from catfish (*Clarias gariepinus*) consumption (μg/kg bw-1week-1).

Metal	Concentration (μg/g ww)	MWI (μg/kg bw^-1^week^-1^)	PTWI (μg/kg bw^-1^week^-1^)
Al	1.12E+01	1.31E+01	2.00E+03
As	1.44E-02	1.68E-02	1.50E+01
Cd	5.92E-04	6.89E-04	2.50E+00
Co	3.26E-02	3.79E-02	7.0 E+2**
Cr	2.81E-01	3.27E-01	2.33E+01
Cu	5.24E-01	6.11E-01	3.50E+03
Fe	2.09E+01	2.43E+01	5.60E+03
Mn	4.87E-01	5.67E-01	9.80E+02
Ni	1.22E-01	1.42E-01	1.10E+01
Pb	9.84E-03	1.15E-02	*
V	2.97E-02	3.45E-02	1.40E+01
Zn	8.56E+00	9.97E+00	7.00E+03

Key: PTWI—Provisional Tolerable Weekly Intakes,

*PTWI withdrew,

MWI—Metal Weekly Intake,

** no PTWI, MTWI.

The PTWI for Cd, Cr, Cu, Ni, and Zn, per kilogram bodyweight (kgbw) are 2.5μg, 23.3μg, 500μg, 11μg and 70μg, respectively.

#### 3.9.2 Assessment of non-carcinogenic and carcinogenic health risk

Nine trace metals (Cd, Cr, Cu, Fe, Ni, Pb, Zn, Co, V), and arsenic were assessed for non-carcinogenic target risk, and four trace metals (Cd, Pb, Cr, Ni) and Arsenic were assessed for carcinogenic target risks. The non-carcinogenic health risk was assessed using the target hazard quotient (THQ) results (Table 10). The non-carcinogenic assessment found none of the assessed metals and a metalloid is likely to contribute to non-carcinogenic health effects over a lifetime exposure (70 years). The total combined THQ was < 1, which indicates that the combined effect of these metals towards non-carcinogenic effects is low.

**Table 10:** Non-carcinogenic health risk effects (THQ) and the carcinogenic target risk (TR).

	Non-carcinogenic risk				Carcinogenic risk	
Metal	Wet Conc	CDI	RfD	THQ	SF	TR
	μg/g (ww)	μg/kg bw day	μg/kg bw day		kg bw day/μg	
Cd	5.92E-04	9.8E-05	1	9,80E-05	6.10E-03	6.0E-07
Pb	9.84E-03	1.6E-03			8.50E-06	1.4E-08
Al	1.12E+01	1.9E+00	285	6.54E-03		
V	2.97E-02	4.9E-03	2	2.46E-03		
Cr	2.81E-01	4.7E-02	2,17	2.14E-02	5.00E-04	2.3E-05
Mn	4.87E-01	8.1E-02	140	5.76E-04		
Fe	2.09E+01	3.5E+00	700	4.95E-03		
Co	3.26E-02	5.4E-03	100	5.40E-05		
Ni	1.22E-01	2.0E-02	11.7	1.73E-03	8.40E-04	1.7E-05
Cu	5.24E-01	8.7E-02	500	1.74E-04		
Zn	8.56E+00	1.4E+00	300	4.73E-03		
As	1.44E-02	2.4E-03	1.5	1.60E-03	1.50E-03	3.6E-06
Combined THQ (HI)				4.43E-02		4.42E-05
Tolerable level				<1		10^−6^ to 10^−4^
Unacceptable				>1		>10^−4^
Reference guideline				[65]		

Key: CDI—Chronic Daily intake, RfD—Reference Dose, THQ—Target Hazard Quotient, SF—Slope Factor, TR—Target Risk. USEPA—United States Environmental Protection Agency, μg/kgbw^-1^day–microgram per kilogram body weight per day and kg/bw/day/μg–Kilogram body weight per day per microgram.

Concerning carcinogenic effects, the carcinogenic target risk factors (TR) range from 1.4 E-8 to 2.3 E-5 for the four trace metals and arsenic analyzed (Cd, Pb, Cr, Ni, As). Results fall within the acceptable range of E-5. The highest risks are observed for Cr and Ni, which are within the acceptable limit. Cr and Ni are also the elements with the highest enrichment and sediments were classified as moderately polluted regarding these elements. The concentrations of Cr and Ni in the catfish were generally higher than other trace metals, indicating that the source, dispersion, and bioaccumulation require further investigation.

## 4. Conclusion and recommendations

This study was conducted to provide information on the pollution status of the studied sites hypothesizing that anthropogenic activities carried upstream pollute sediments with trace metals and arsenic downstream, and assessed the effects on biota represented by catfish. Our PLI findings suggest polluted sediment, while individual EF results indicate that sediments are moderately polluted with Al, Cr, Fe, Mn and V but the levels of As, Cd, Co, Cu, Pb, and Zn were within the Interim Sediment Quality Guideline of the USEPA and reflect the natural background. The lgeo results suggest that the source of the trace metals is geogenic and results for areas with high anthropogenic and little human activities produced comparable levels of trace metals. The risk indices indicate low risks both to the catfish and the catfish-consuming population along the study areas. The Carcinogenic Target Risk, Metal Weekly Intake, and Target Hazard Quotient, when compared to the established references, all suggested that catfish were not exposed to lethal doses of trace metals; hence, the local human population would take no risk from consuming catfish even daily. The two major limitations this study suffered were the low number of samples used as well as only catfish muscle tissues for trace metal analysis. Other researchers can work on the same topic but increase the sample size, and analyze different catfish organs for trace metal levels and the risk to biota.

As a preventive measure, continuous public health education to the community on the sources and health effects of trace metals, and a continued monitoring program of trace metals in the environment and biota is suggested.

## Supporting information

S1 Table Table of wet and dry weight. (PDF)
