# Supplementary material for: Status of trace metals and arsenic in sediments and catfish muscles (Clarias gariepinus) from the Eastern Tanzanian basin
Source: PLoS One. 2024 Aug 29;19(8):e0306335. doi: 10.1371/journal.pone.0306335 (PMC11361695; doi:10.1371/journal.pone.0306335)
Supplement: S1 Table — (PDF) [file pone.0306335.s001.pdf]

Supplementary table 1

S/N	Site	Wet wt	Dry wt	% Dry wt	% Wet wt
**1**	Matandu	7,44	1,35	18,15	81,85
**2**	Matandu	4,71	0,98	20,81	79,19
**3**	Matandu	4,82	0,89	18,46	81,54
**4**	Matandu	3,28	0,77	23,48	76,52
**5**	Matandu	5,42	1,34	24,72	75,28
**6**	Matandu	4,57	0,88	19,26	80,74
**7**	Matandu	8,92	1,98	22,20	77,80
**8**	Matandu	4,62	0,71	15,37	84,63
**9**	Rufiji	11,05	2,14	19,37	80,63
**10**	Rufiji	14,31	2,94	20,55	79,45
**11**	Rufiji	13,24	2,92	22,05	77,95
**12**	Rufiji	5,61	1,2	21,39	78,61
**13**	Rufiji	13,56	2,85	21,02	78,98
**14**	Rufiji	9,13	1,75	19,17	80,83
**15**	Rufiji	12,1	2,82	23,31	76,69
**16**	Rufiji	7,67	1,35	17,60	82,40
**17**	Saadani	10,6	2,28	21,51	78,49
**18**	Saadani	7,89	1,78	22,56	77,44
**19**	Saadani	6,75	1,39	20,59	79,41
**20**	Saadani	6,78	1,24	18,29	81,71
**21**	Saadani	8,45	1,72	20,36	79,64
**22**	Saadani	6,45	1,04	16,12	83,88
**23**	Saadani	12,58	2,69	21,38	78,62
**24**	Saadani	11,9	1,91	16,05	83,95
**25**	Upper Ruvu	4,76	1,01	21,22	78,78
**26**	Upper Ruvu	6,03	1,09	18,08	81,92
**27**	Upper Ruvu	5,15	1,11	21,55	78,45
**28**	Upper Ruvu	8,44	1,9	22,51	77,49
**29**	Upper Ruvu	4,88	1,06	21,72	78,28
**30**	Upper Ruvu	10,05	2,38	23,68	76,32
**31**	Upper Ruvu	8,23	1,83	22,24	77,76
**32**	Upper Ruvu	8,75	1,95	22,29	77,71
**33**	Lower Ruvu	3,77	0,68	18,04	81,96
**34**	Lower Ruvu	4,38	0,89	20,32	79,68
**35**	Lower Ruvu	4,62	0,93	20,13	79,87
**36**	Lower Ruvu	4,58	0,96	20,96	79,04
**37**	Lower Ruvu	5,9	1,05	17,80	82,20
**38**	Lower Ruvu	3,32	0,76	22,89	77,11
**39**	Lower Ruvu	7,32	1,64	22,40	77,60
**40**	Lower Ruvu	7,91	1,58	19,97	80,03
	Average				79,51
